# Risk factors for ACL revision failure and optimum graft size for revision anterior cruciate ligament reconstruction

**DOI:** 10.1007/s00590-025-04381-7

**Published:** 2025-06-19

**Authors:** Victor Yan Zhe Lu, Dennis Hei Yin Lee, Simon Ho Yin Tsui, Thomas Chun Hei Lo, Wai Wang Chau, Annubrat Kumar, Michael Tim Yun Ong, Patrick Shu Hang Yung, Jonathan Patrick Ng

**Affiliations:** Chinese University of Hong Kong, Sha Tin, Hong Kong China

**Keywords:** Anterior cruciate ligament, Rupture, Sports injury

## Abstract

**Introduction:**

Graft re-rupture is a devastating complication after revision ACLR surgery. The literature regarding the risk factors of graft re-rupture is sparse and not definitive. Studies have suggested that a smaller graft diameter is associated with poorer outcomes after primary ACLR, however there is a paucity of literature regarding the effects of graft size on revision ACLR outcomes. This study aims to determine the risk factors for graft re-rupture after revision ACLR, and investigate the optimum graft diameter for revision ACLR.

**Methods:**

The records of all patients who underwent revision ACLR from 2013 to 2021 were reviewed. Data collected included patient demographics, operative variables, and demographic details. To determine the optimal graft diameter, receiver operating characteristic (ROC) analysis was performed. Associations between re-rupture rate and return to pivoting sport, intra-articular knee pathologies, and graft diameter were assessed using contingency tables. Data were examined using univariable logistic regression models to explore the association between graft re-rupture after revision ACLR and prognostic variables. Co-variates with a *p* value *p* < 0.100 were included in a multivariable logistic regression model to identify independent associations with graft re-rupture.

**Results:**

In total, 132 revision ACLR were identified with a mean follow-up time of 3.22 ± 3.26 years. The graft re-rupture rate was 16.7% (*n* = 22). There were 91 (68.9%) males and 41 (31.1%) female with a mean age of 27.4 years (range 17.3–50.8 years) at revision. 87.9% (*n* = 116) were involved in one or more types of pivoting sports. Kaplan–Meier analysis showed that the mean survival time for revision ACL grafts was 148 months (95% CI 130–166). The mean graft diameter during revision ACLR was 9.26 mm (range 7.0–10.5 mm) and mean graft length was 43.6 mm (range 22.0–60.0 mm). No associated procedure such as anterolateral (ALL) reconstruction were performed. At the time of revision ACLR, MRI detected concomitant knee pathologies: medial meniscus pathology (*n* = 45; 34.1%), lateral meniscus pathology (*n* = 41; 31.1%), chondral pathology (*n* = 26; 19.7%). None were associated with an increased rate of re-rupture. Risk factors determined by the multivariable logistic regression model were graft diameter < 9 mm (OR: 3.873; 95% CI 1.128–13.293; *p* = 0.031) and return to pivoting sport after revision ACLR surgery (OR: 4.105; 95%CI 1.008–16.721; *p* = 0.049).

**Conclusion:**

A graft diameter < 9 mm and return to pivoting sports after revision ACLR are risk factors for graft re-rupture. Meniscus pathology and chondral lesion were not associated with graft re-rupture. The findings of this study can be used to improve revision ACLR results for patients, but needs to be expanded in multi-centre trials with larger sample sizes.

## Introduction

Anterior cruciate ligament (ACL) tears are a common sports injury, especially in pivoting sports that cause high-impact loading on the knee, such as football, basketball, and volleyball. ACL tears are commonly associated with intra-articular pathologies such as chondral lesions and meniscus tears [1], which can contribute to the development of knee osteoarthritis (OA) [2]. Early surgical treatment can prevent the development of such lesions and subsequent degenerative changes [2]. Surgical treatment is also warranted for those with continuous instability symptoms or a desire to return to sports [3]. 

Surgical treatment of ACL tears over the past two decades have relied on ACL reconstruction (ACLR) with the use of a graft to stabilise the knee joint [2]. Graft failure remains one of the devastating complications of ACLR. Patients are faced with the prospect of a revision ACLR as well as the physical and mental challenges of an additional rehabilitation program. As more people engage in high-impact pivoting sports, the rate of revision ACLRs has been steadily rising [1].

Compared with data on primary ACLR, studies reporting outcomes after revision ACLRs remain sparse [4]. Studies on revision ACLR usually describe the surgical technique; those that report clinical outcomes have small sample sizes and use patient cohorts with a broad age range. Nevertheless, studies show that the clinical outcomes are poorer than those after a primary ACLR [4–6]. This could be due to the increased complexity of revision ACLR surgery due to limited graft choice and difficult tunnel placement, increased incidence in intra-articular pathologies during surgery, and development of post-traumatic OA [7].

An important difference between primary and revision ACLR is the four-fold increase in graft failure rates [4]. The Multi-centre ACL Revision Study (MARS), a multi-centre, multi-surgeon prospective study, reported a 3.3% re-rupture rate at two year follow-up. Data from the Kaiser Permanente ACLR Registry reported a 4.3% re-rupture rate at 2.6 years follow-up. The risk factors for multiple graft failures are poorly understood. Intra-articular lesions have been shown to be more prevalent after revision surgery, which leads to poorer outcomes and increased risk of re-rupture [8]. Other reported risk factors for re-rupture include return to high-impact pivoting sports [9], a higher pre-operative pivot shift grade [10], and young age [8]. These factors are interrelated since younger patients are more likely to return to high-risk sports after primary ACLR [9], however there is limited data on the behaviour of younger patients after revision ACLR.

Graft diameter is an important predictor of revision rates in ACLR surgery. Although the optimum graft size for ACLR remains undefined, studies investigating graft size in primary ACLR suggest that a graft size ≥ 8 mm is correlated with a lower incidence of graft failure and subsequent revision ACLR [11–14]. However, data regarding the optimum graft size to prevent re-rupture after revision ACLR remains sparse.

This study aimed to determine the risk factors of re-rupture after revision ACLR, and to evaluate the effect of graft diameter on re-rupture rate after revision ACLR.

## Methods

There was a retrospective multi-centre cohort study performed in Hong Kong. Patients were included if they underwent revision ACLRs between January 2013 and January 2023. Patients were identified using the Clinical Data Analysis and Reporting System (CDARS), an electronic health records database covering all public hospitals and clinics in Hong Kong. This captures inpatient data from 80 to 90% of the population [15]. To ensure confidentiality, all patients were anonymized in CDRAS. This study gained ethical approval from the Clinical Research Ethics Committee (CREC) and was performed according to the STROBE guidelines [16].

Patients were excluded if they had previous meniscectomy, previous ACL reconstruction with lateral extra-articular tenodesis (LET) procedure, inflammatory arthritis in the affected knee, or inability to follow the rehabilitation program. Patient characteristics captured include age, BMI, gender and type of sport at injury. For both primary ACLR and revision ACLR, operative variables (graft choice, graft size, fixation method, and tibial slope), radiographic details (tunnel position, concomitant knee pathologies) were obtained, serving as the basis for the identification of risk factors and covariables in this study. Where present, concomitant knee pathologies were direct visualised during revision ACLR, and surgical reports were scrutinised to obtain data. Descriptive statistics such as percentage of graft re-ruptures and return to sports rates were calculated as percentages. Mean and standard deviation (SD) were provided where appropriate. Comparisons between two independent groups were performed using Student’s t-test for parametric data or Mann–Whitney U test for nonparametric data. Associations between re-rupture rate and return to pivoting sport, intra-articular knee pathologies, and graft diameter were assessed using contingency tables. Results were presented using the *χ*2 statistic with 95% confidence intervals. To determine the optimal graft diameter, receiver operating characteristic (ROC) analysis was performed [17]. The area under the curve (AUC) was calculated to find a criterion value that is best able to distinguish between those with or without a re-rupture.

Graft failure is defined in the 2022 European Society of Sport Traumatology, Knee Surgery and Arthroscopy (ESSKA) consensus statement as ‘abnormal knee function associated with a previous primary reconstruction’ [18]. For this study, revision ACL graft failure was defined as graft re-rupture shown radiographically through magnetic resonance imaging (MRI) scans, or clinically after a traumatic episode leading to a previous stabilised knee to become unstable, or continuing instability post-reconstruction. Not all re-ruptures underwent re-revision surgery (3rd ACL reconstruction).

Data were examined using univariable logistic regression models to explore the association between graft re-rupture after revision ACLR and prognostic variables. Assessed criteria include age, gender, BMI, presence of concomitant knee pathologies, tibial slope, pivot shift grade, graft diameter, and return to pivoting sport. Co-variates with a *p* value *p* < 0.100 were included in a multivariable logistic regression model to identify independent associations with graft re-rupture. Survivorship analysis was performed using the Kaplan–Meier estimate. The log rank test was used to determine if there is a difference between two populations in the probability that graft re-rupture occurred [19]. Statistical analysis was performed using IBM SPSS version 28.0.1. Statistical significance was set at *p* < 0.05.

## Results

In total, 132 revision ACLR were identified with a mean follow-up time of 3.22 ± 3.26 years (Table 1). There were 91 (68.9%) males and 41 (31.1%) female with a mean age of 23.1 years (range 15–48) at their primary ACLR reconstruction, and 27.4 years (range 17.3–50.8 years) at revision. Pivoting sports such as basketball (*n* = 40; 30.3%), football (*n* = 48; 36.4%), volleyball (*n* = 7; 5.30%), rugby (*n* = 14; 10.61%), badminton (*n* = 1; 0.76%), tennis (*n* = 2; 1.52%), judo (*n* = 1; 0.76%), handball (*n* = 1; 0.76%), hockey (*n* = 1; 0.76%) squash (*n* = 1; 0.76%) accounted for 87.9% (*n* = 116) of preinjury sports.

**Table 1:** Patient demographics and operative details

Variable	Mean (± SD or range) or n (%)
Age at primary ACLR (years)	23.1 (range 15–48)
Age at revision ACLR (years)	27.4 (range 17.3–50.8)
BMI	23.11 ± 3.23
Mean follow-up time (years)	3.22 ± 3.26
Gender	
Male	91 (68.9%)
Female	41 (31.1%)
Pivoting Sport	
Basketball	40 (30.3%)
Football	48 (36.4%)
Volleyball	7 (5.3%)
Rugby	14 (10.6%)
Badminton	1 (0.8%)
Tennis	2 (1.5%)
Judo	1 (0.8%)
Handball	1 (0.8%)
Hockey	1 (0.8%)
Squash	1 (0.8%)
Non-Pivoting Sport	
Dancing	1 (0.8%)
Gymnastics	2 (1.5%)
Rock Climbing	1 (0.8%)
Taekwondo	1 (0.8%)
Rope Jumping	1 (0.8%)
Hiking	1 (0.8%)
Running	2 (1.5%)
Golf	1 (0.8%)
Femoral tunnel size (mm)	11.1
Tibial tunnel size (mm)	11.3
Pivot Shift Grade	
1	6 (4.5%)
2	50 (37.9%)
3	54 (40.9%)
Primary ACLR	
Graft choice	BPB: 26 (19.7%); HS: 101 (76.5%)
Femoral fixation choice	EB: 1 (0.8%); S: 89 (67.4%); S+IF: 6 (4.5%); IF: 17 (12.9%)
Tibial fixation choice	S: 6 (4.5%); IF: 112 (84.8%); S+IF: 3 (2.3%)
Notch size (mm)	20.6
Knee pathology	MM: 28 (21.2%); LM: 44 (33.3%); Chondral: 9 (6.8%)
Return to pivoting sport	Yes: 98 (74.2%); No: 22 (16.7%)
Revision ACLR	
Graft choice	BPB: 77 (58.3%); HS: 48 (36.4%); Achilles: 2 (1.5%)
Femoral fixation choice	IF: 95 (72.0%); S: 17 (12.9%); S+IF: 2 (1.5%)
Tibial fixation choice	IF: 132 (100%)
Number of stages	One-stage: 126 (95.5%); Two-stage: 6 (4.5%)
Knee pathology	MM: 45 (%); LM: 41 (31.1%); Chondral: 26 (19.7%)
Graft diameter (mm)	9.26 (range 7.0–10.5)
Return to pivoting sport	Yes: 54 (40.9%); No: 44 (33.3%)
Re-rupture	22 (16.7%)

ACLR, anterior cruciate ligament reconstruction; EB, endobutton; IF, interference screw; S, suspension; MM, medial meniscus; LM, lateral meniscus; BPB, bone–patellar tendon–bone; HS, hamstring

### Operative details

On average, revision ACLR was performed 4.43 years after primary ACLR. Twenty-six patients were treated with primary bone–patellar tendon–bone (BPB) grafts, of which, two (7.7%) were treated with revision BPB grafts, twenty three (88.5%) were treated with revision hamstring grafts, and one (3.8%) was treated with revision Achilles tendon graft. One hundred and six patients were treated with a primary hamstring graft, of which twenty two were treated revision hamstring grafts (20.8%), eighty-one were treated with revision BPB grafts (76.4%), and three were treated with revision Achilles tendon grafts (2.8%). The most common type of graft used in primary ACLR and revision ACLR were hamstring tendon grafts (*n* = 102; 77.3%) and bone–patellar tendon–bone (BPB) grafts (*n* = 77; 58.3%), respectively. The selection of graft for revision surgery was dependent on multiple factors, such as patient satisfaction with the previous graft, the need to kneel postoperatively, return to sports, muscle strength, patient’s request to restrict surgery to one knee, and any complications at the donor site during the primary ACLR. The mean graft diameter during revision ACLR was 9.26 mm (range 7.0–10.5 mm) and mean graft length was 43.6 mm (range 22.0–60.0 mm). At the time of revision ACLR, concomitant knee pathologies were detected in several patients: medial meniscus pathology (*n* = 45; 34.1%), lateral meniscus pathology (*n* = 41; 31.1%), chondral pathology (*n* = 26; 19.7%). None were associated with an increased rate of re-rupture (Table 2). Femoral and tibial tunnel position was in a satisfactory position for re-drilling in 98 patients; 34 patients required a new tunnel to be created. 40.9% (*n* = 54) of patients had a grade III pivot shift. During revision ACLR, an interference screw was used for femoral fixation in 72% of grafts, and tibial fixation for all grafts. Six patients received a two-stage revision ACLR, none of which had a graft re-rupture; the rest had a single-stage revision ACLR. No augmentation procedures such as anterolateral ligament (ALL) reconstruction or lateral extra-articular tenodesis (LET) were performed.

**Table 2:** Relationship between Graft re-rupture and knee pathologies at time of revision ACLR surgery

		Pathology present at revision ACLR	No pathology at revision ACLR	OR for Re-rupture (95% CI)	*P* value*
Medial Meniscal Pathology	Graft re-rupture	7	15	0.884 (0.332–2.355)	0.805
	No re-rupture	38	72		
Lateral Meniscal Pathology	Graft re-rupture	5	17	0.605 (0.207–1.769)	0.355
	No re-rupture	36	74		
Chondral Pathology	Graft re-rupture	7	15	2.235 (0.802–6.225)	0.802
	No re-rupture	19	91		

OR, odds ratio; CI, confidence interval; ACLR, anterior cruciate ligament reconstruction

*From *x*^2^ statistic

### Graft re-rupture

**Fig. 1:**
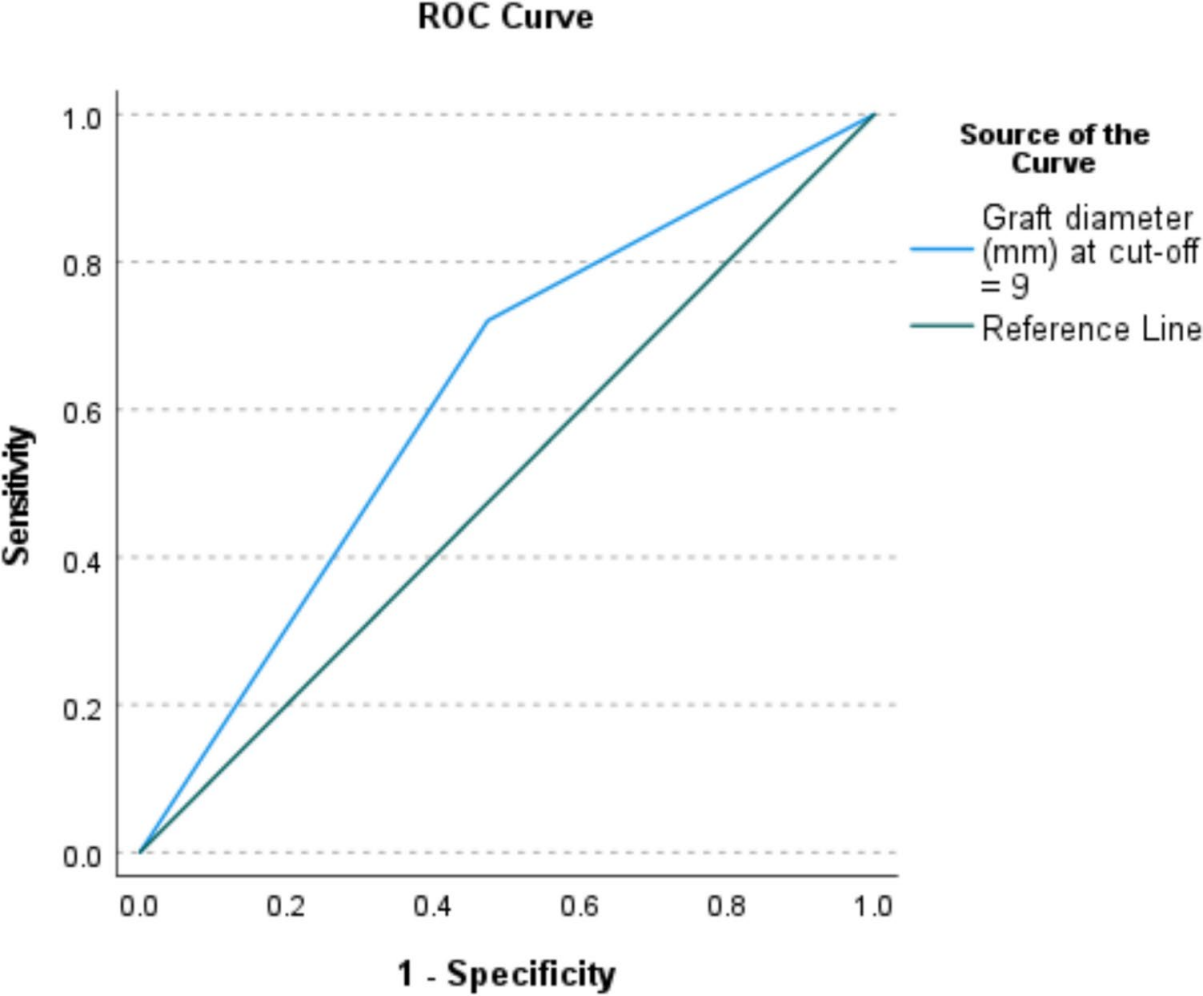
Receiver-operating characteristic (ROC) analysis to determine optimum graft diameter

The graft re-rupture rate was 16.7% (*n* = 22), 50% of which occurred in hamstring tendon grafts and 50% in BPB grafts. Graft choice had no significant impact on re-rupture rates (*χ*^2^ = 1.96; *p* = 0.375). 11.4% (*n* = 15) of the revision ALCRs were revised a second time. Those who had a re-rupture had a significantly higher rate of return to pivoting sport after revision ACLR (*n* = 16; 72.7%), than those without a re-rupture (*n* = 38; 34.5%) (*χ*^2^ = 7.5; *p* = 0.006) (Table 3). Graft diameters ranged from 7.0 mm to 10.0 mm. A cutoff value of 9 mm was found to be the optimum graft diameter during revision ACLR (AUC: 0.63; *p* = 0.049) (Table 4) (Fig. 1). The re-rupture rate was 27% in grafts < 9 mm, 32.3% in grafts < 8.5 mm, 35.0% in grafts < 8.0 mm, and 40.0% in grafts < 7.5 mm. The only patient with a graft diameter of 7.0 mm experienced a re-rupture. Compared to patients without ACL re-rupture, patients who experienced a re-rupture had a significantly higher number of grafts with diameter < 9 mm (45.5% versus 24.5%; *χ*^2^ = 4.1; *p* = 0.043). Risk factors determined by the multivariable logistic regression model were graft diameter < 9 mm (OR: 3.873; 95% CI 1.128–13.293; *p* = 0.031) and return to pivoting sport after revision ACLR surgery (OR: 4.105; 95%CI 1.008–16.721; *p* = 0.049) (Table 5). Age had an impact on the return to pivoting sport after revision ACLR. 71.4% of those under 18 returned to pivoting sport, whilst 38.4% of those aged 18 or over returned to pivoting sport.

**Table 3:** Rates of Return to Pivoting Sport after ACLR Stratified by Third ACL Injury

	Third ACL injury (*n* = 22)	No further ACL injury (*n* = 110)
Primary ACLR	18 (81.8%)	80 (72.7%)
Revision ACLR	16 (72.7%)	38 (34.5%)

**Table 4:** Optimum graft diameter during revision ACLR surgery

Cutoff value	AUC (95% CI)	SE	*P* value
< 8	0.53 (0.38–0.68)	0.08	0.275
< 9	0.63 (0.48–0.77)	0.09	0.049*
< 10	0.61 (0.47–0.74)	0.07	0.101

ACLR, anterior cruciate ligament reconstruction; AUC, area under curve; CI, confidence interval; SE, standard error

**Table 5:** Univariable and Multivariable logistic regression analyses

*N* = 132	Univariable			Multivariable		
	Re-rupture (%; *n* = 22)	No re-rupture (%; *n* = 110)	*P* value	Odds Ratio	95% CI	*P* value
MM injury at revision ACLR	7 (31.8)	38 (34.5)	0.288			
LM injury at revision ACLR	5 (22.7)	36 (32.7)	0.108			
Chondral injury at revision ACLR	7 (31.8)	19 (17.3)	0.261			
Tibial slope (degrees)	13.14	12.61	0.489			
Pivot shift grade	Grade 1 (*n* = 0); Grade 2 (*n* = 7); Grade 3 (*n* = 13); Unknown (*n* = 2)	Grade 1 (*n* = 6); Grade 2 (*n* = 43); Grade 3 (*n* = 41); Unknown (*n* = 20)	0.067	2.283	0.779–6.711	0.132
Graft < 9 mm diameter	10 (45.5)	27 (24.5)	0.032	3.873	1.128–13.293	**0.031**
Return to pivoting sport after revision ACLR	16 (72.7)	38 (34.5)	0.029	4.105	1.008–16.721	**0.049**
Age < 18 years old	7 (31.8)	12 (10.9)	0.107			
BMI	23.46	23.90	0.545			
Male	12 (54.5)	79 (71.8)	0.110			

ACLR, anterior cruciate ligament reconstruction; MM, medial meniscus; LM, lateral meniscus; CI, confidence interval; BMI, body mass index

**Fig. 2:**
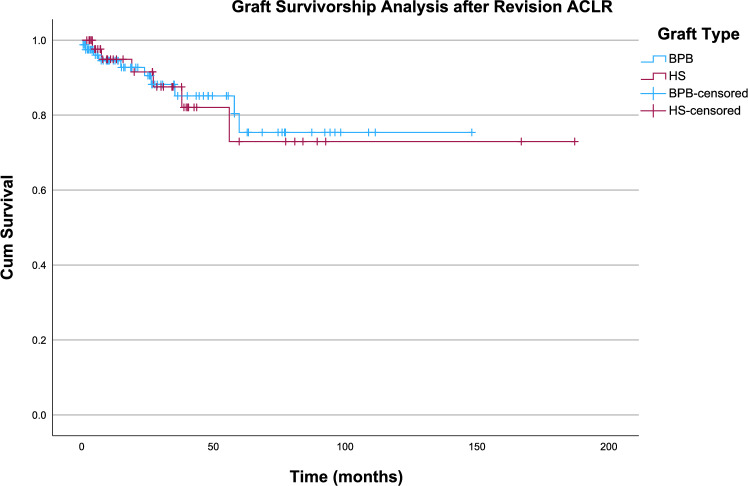
Kaplan–Meier analysis comparing survivorship between BPB grafts and hamstring grafts

**Fig. 3:**
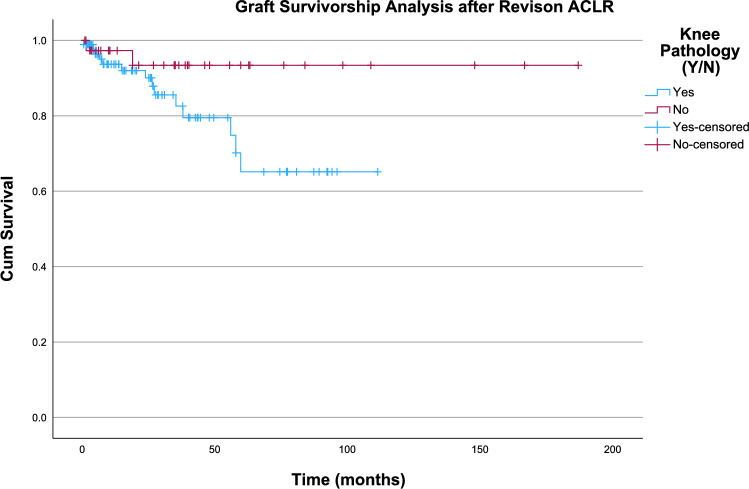
Kaplan–Meier analysis comparing graft survivorship between those with concomitant knee pathologies and those without

Survivorship analysis was performed using the Kaplan–Meier estimator. The mean survival time for revision ACL grafts was 148 months (95% CI 130–166). Log rank test showed that there is no difference in revision ACL graft survivorship between those that received BPB tendon versus those that received hamstring tendon (*χ*^2^ = 0.003, *p* = 0.957) (Fig. 2). There was also no significant difference in revision ACL graft survivorship between those that had concomitant knee pathologies and those that did not (*χ*^2^ = 3.177, *p* = 0.075) (Fig. 3).

## Discussion

The results of this study suggest that a graft diameter of 9 mm or less, and return to pivoting sport after revision ACLR surgery are risk factors for graft re-rupture. Both hamstring and BPB tendon grafts were equally susceptible to re-rupture in this study. Although there is only a small amount of literature on this topic, the re-rupture rate of 16.7%, and re-revision rate of 11.4% in this study is high compared to other cohorts. Studies in the USA report a re-revision rate ranging from 3.3 to 13.7% [4, 8, 20, 21]. Webster et al. reported a 15% graft re-rupture rate in a population of 128 from Australia [9]. Noyes et al. performed a study in 2001, reporting a 24% re-rupture rate after revision ACLR, but a 7% re-rupture rate after primary ACLR [22]. In a systematic review of twenty-one studies evaluating the outcomes of revision ACLR, the objective failure rate was 13.7% ± 2.7%. However, their definition of ‘failure’ included many parameters in addition to graft re-rupture, such as a pivot shift grade of 2 + or 3 + and side-to-side difference of > 5 mm. Nevertheless, reoperation rates after revision ACLR is not a commonly reported figure in most studies [4]. Over a five year follow-up period, a study including nearly 18,000 patients from the Swedish National ACL Registry reported a revision rate of 9.8% for males and 22.0% revision rate for females [23]. However, the reoperation rate after revision ACLR was not specified, and the re-injury rate, which is likely to be higher than the reoperation rate, was also not reported.

Studies have suggested that young age is a risk factor for re-rupture [9, 24–26]. Despite not reaching statistical significance in the univariable model (*p* = 0.107), there was a three-fold increased rate of re-rupture in those under 18 versus older patients (31.8% vs 10.9%). Magnussen et al. reported a < 1% revision rate in those aged twenty or older, compared to a 16.5% revision rate in those aged under twenty [14], attributing the finding to increased activity levels in younger patients. Kamien et al. reported a higher failure rate in those aged twenty-five or below (25% versus 6%) [27]. Young age is likely a proxy for increased return to sport, especially pivoting sport which cause higher-impact loading on knees and increased risk of ACL injury [28]. A Norwegian study on 210 patients found that those who returned to pivoting sport were significantly younger than those who returned to non-pivoting sport [2]. The 2022 ESSKA consensus statement suggested those with a high sports activity score are more suitable for ACL revision surgery than conservative treatment [29].

Pivoting sport was a risk factor for ACL re-rupture in our study, however it is important to note that this covariable had borderline statistical significance (*p* = 0.049). After primary ACLR, there was a high rate of return to pivoting sport in patients who suffered a third ACL injury and those who did not (81.8% versus 72.7%). The return to sport rate decreased for the whole population after revision ACLR, which is consistent with other studies [9, 30]. However, patients with a third ACL injury had a similarly high rate of return to pivoting sport (81.8% vs 72.7%), whilst those without a third ACL injury had a much lower rate (72.7% vs 34.5%). Webster et al. also found that return to sport is associated with re-rupture (*x*^2^ = 5.5; *p* = 0.019). Pivoting sports involves rapid directional changes in movement, which puts significant stress on the reconstructed ligament, especially if the surrounding tissues have not fully healed. It is important that knee stability and neuromuscular control are adequately assessed before returning to sports. Rehabilitation timelines would be a useful variable, however this was not collected in this study.

Although a higher pivot shift grade was not a risk factor for re-rupture in the multivariable analysis (p = 0.132), other studies have demonstrated an increased risk of revision surgery in these patients [31–33]. Even if patients with a re-rupture were excluded, those with a higher pivot shift grade have poorer patient reported outcome measures (PROMs) [34], perhaps due to an increased incidence of knee OA. A higher pivot shift grade is associated with generalised ligamentous laxity, which is correlated with an increased risk of knee OA [31]. Studies have suggested using augmentation procedures in such patients to improve clinical outcomes, such as ALL reconstruction [33], or lateral extra-articular tenodesis [35].

Revision ACLR is technically challenging, not least due to difficult tunnel positioning, limited graft harvesting options, and patient factors. Furthermore, the increased incidence of intra-articular lesions during revision surgery such as meniscus pathology, chondral lesions [7], and the development of post-traumatic OA can further complicate surgery and rehabilitation. The MARS group reported that patients with medial meniscus pathology at revision ACLR had a significant lower KOOS score and were > 2 times more likely to have subsequent surgery (OR 2.2; *p* = 0.005), compared to those who had an intact medial meniscus (OR: 1.45–1.59; *p* ≤ 0 0.03) [36]. Webster et al. reported that medial meniscus injury was a risk factor for graft re-rupture (*χ*^2^ = 5.2, *P* = 0.02) [9]. However, amongst the patients with graft re-rupture, 70% had medial meniscus injury. In this study, 38% of those with a re-rupture had a medial meniscus pathology, whilst 34.5% of patients without a re-rupture had medial meniscus pathology. No association between medial meniscus injury and graft re-rupture was found in this study. The association between lateral meniscus pathology and chondral lesions and multiple revision surgeries is less robust. The MARS group reported that patients with lateral meniscus injury before primary ACLR affected 2 year PROMs [37]. Webster et al. found no relationship between lateral meniscus injury and functional scores, and no relationship between chondral lesions and graft re-rupture [9]. The MARS group however reported that chondral pathology is a risk factor for subsequent surgery within six years of primary ACLR [36]. The current study found no association between any intra-articular pathology and graft re-rupture.

Regarding primary ACLR surgery, studies have suggested that a graft size of ≥ 8 mm is associated with better outcomes [11–14, 38]. Magnussen et al. reported a 1.7% revision rate in grafts ≥ 8 mm, but a 13.6% revision rate in grafts < 7 mm [14]. Park et al. [11] and the MOON cohort study [13] reported no revisions in those with grafts ≥ 8 mm. One study that utilised a mixture of BPB and hamstring grafts reported no difference in graft failure rates between different graft sizes (< 8 mm vs > 8 mm) [39]. There is however limited data regarding the optimal graft size for revision ACLR surgery. Our study suggests a graft size of ≥ 9 mm is associated with better outcomes. However, it is important to note that the AUC value of 0.63 is relatively close to 0.5, which indicates low discriminative power and limited predictive value. Furthermore, the p value of 0.049 suggests borderline statistical significance.

The majority of evidence on this topic concludes that a smaller graft size is associated with a higher failure rate [3]. This trend was also depicted in this study, with a 0.5 mm increase in graft diameter affecting re-rupture rates. Biomechanical studies have shown a significant increase in graft tensile strength with increasing diameter [40]. A biomechanical study performed by Boniello et al. showed that only grafts with a diameter of 8 mm or more had a tensile strength that reached the commonly accepted value of 4000N. Mariscalco et al. showed that graft sizes of 8 mm or less had a 0% failure rate, whilst the failure rate with graft diameters over 8 mm was 18.3% in adults [13]. However, little difference in critical strength threshold was seen between different graft types of small diameters [41]. The ideal graft diameter needed to avoid failure is unclear. Perhaps graft diameter should be a continuous variable dependent on demographic factors such as patient size and weight.

In addition to the retrospective nature of this study, limitations include the lack of contralateral ACL injury data, which could suggest a high baseline rate of ACL injury in this population. Time from revision ACLR surgery to return to pivoting sport was not collected, which could influence the re-rupture rate. Beischer et al. found that those who return to sport nine months after ACLR surgery have a seven fold increased rate of a subsequent ACL injury [24]. Other confounding factors such as ethnicity were unaccounted for, which could have an effect on re-rupture rates [42]. Revision grafts were heterogenous and technical details such as surgeon technique, tunnel placement, and fixation methods were not controlled. The mechanism of graft re-rupture was not reported, which could help explain why different graft sizes result in different re-rupture rates. This study lacked data on rehabilitation adherence and PROMs after revision ACLR, (e.g. KOOS, EQ-5D, and IKDC), which have been shown to be predictors of re-rupture [25]. Pre-op and post-op knee laxity scores were not measured (e.g. KT-1000), and data regarding meniscus treatments were not collected, which could have influenced the outcomes. The possibility of differing risk factors depending on follow-up time was not explored [26]. The small sample size collected from a single institution limits the internal and external validity of this study. Future studies with large sample sizes are warranted to investigate how graft diameter affects the outcomes of revision ACLR.

## Conclusion

Return to pivoting sports after revision ACLR and a graft diameter < 9 mm are factors significantly associated with re-rupture. There was a three-fold increase rate of re-rupture in those under 18 versus older patients. Meniscus pathology and chondral lesions were not associated with an increased re-rupture rate. Although a graft diameter of ≥ 9 mm was found to be optimal in this study, multi-centre studies with larger numbers of revision ACLR are needed to confirm this finding.

## Data Availability

No datasets were generated or analysed during the current study.
