# Correction: Risk factors for ACL revision failure and optimum graft size for revision anterior cruciate ligament reconstruction

**DOI:** 10.1007/s00590-025-04540-w

**Published:** 2025-10-06

**Authors:** Victor Yan Zhe Lu, James Hong Yin Woo, Dennis Hei Yin Lee, Simon Ho Yin Tsui, Thomas Chun Hei Lo, Wai Wang Chau, Anubrat Kumar, Michael Tim Yun Ong, Patrick Shu Hang Yung, Jonathan Patrick Ng

**Affiliations:** Chinese University of Hong Kong, Sha Tin, Hong Kong China

**Correction to: European Journal of Orthopaedic Surgery & Traumatology (2025) 35:260** 10.1007/s00590-025-04381-7

In the original version of this article, author James Hong Yin Woo and his email address Jameswoo@link.cuhk.edu.hk was missing and now it has been added.

The author’s name Anubrat Kumar was incorrectly written as Annubrat Kumar and e-mail address of the author Anubrat Kumar was incorrectly given as 1155159170@link.cuhk.edu.hk where it should have been Dranubrat5@gmail.com 

